# Coordination of kidney organogenesis by Wnt signaling

**DOI:** 10.1007/s00467-013-2733-z

**Published:** 2014-01-21

**Authors:** Kimmo Halt, Seppo Vainio

**Affiliations:** 1 The Centre of Excellence in Cell-Extracellular Matrix Research, Oulu, Finland; 2 Biocenter Oulu, Oulu, Finland; 3 Laboratory of Developmental Biology, Department of Medical Biochemistry and Molecular Biology, Institute of Biomedicine, University of Oulu, PO Box 5000, 90014 Oulu, Finland

**Keywords:** Wnt signaling, Tubule induction, Nephron segmentation, PCP

## Abstract

Several Wnt proteins are expressed in the embryonic kidney during various stages of development. Gene knockout models and ex vivo studies have provided strong evidence that Wnt-mediated signals are essential in renal ontogeny. Perhaps the most critical factors, Wnt9b and Wnt4, function during the early phase when the cap mesenchyme is induced to undergo morphogenesis into a nephron. Wnt11 controls early ureteric bud branching and contributes to the final kidney size. In addition to its inductive role, later on Wnt9b plays a significant role in the convergent extension of the tubular epithelial cells, while Wnt4 signaling controls smooth muscle cell fates in the medulla. Wnt7b has a specific function together with its likely antagonist Dkk1 in controlling the morphogenesis of the renal medulla. The signal-transduction mechanisms of the Wnts in kidney ontogeny have not been resolved, but studies characterizing the downstream signaling pathways are emerging. Aberrant Wnt signaling may lead to kidney diseases ranging from fatal kidney agenesis to more benign phenotypes. Wnt-mediated signaling regulates several critical aspects of kidney development from the early inductive stages to later steps of tubular epithelial maturation.

## Introduction

**Fig. 1:**
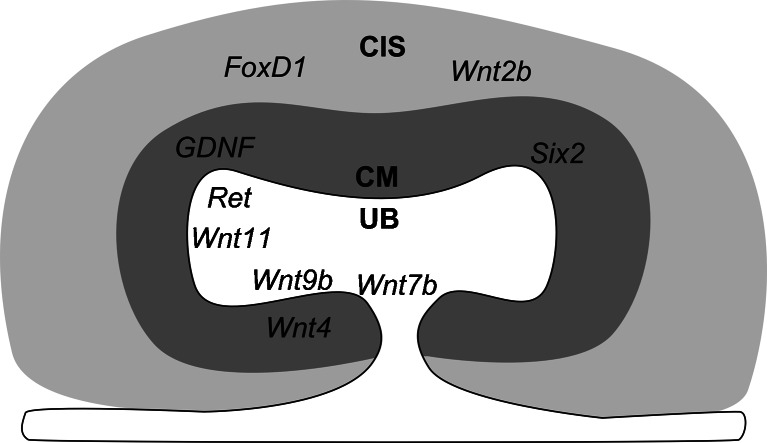
Schematic micrograph of the embryonic kidney. A ureteric bud (UB, *white*) invades the metanephric mesenchyme, which consists of the cap mesenchyme (CM, *dark grey*) and the cortical interstitial mesenchyme (CIS, *light grey*). *Six2* and *FoxD1* transcription factors mark the CM and CIS, respectively. *Wnt9b* is expressed in the UB where its paracrine action induces *Wnt4* expression in the ventral CM. *Wnt11* in the UB tips establishes a positive signaling loop with the GDNF/Ret system to promote UB branching. *Wnt7b* is expressed in the UB where it regulates the development of the renal medulla. The role of *Wnt2b* in the CIS is unknown

The definitive kidney of mammals, the metanephros, appears as a morphologically distinguishable rudiment around midgestation [1]. The early metanephros is composed of the epithelial ureteric bud (UB), an outgrowth from the adjacent Wolffian duct, and the metanephric mesenchyme (MM). The MM is divided into the cap mesenchyme (CM), which is adjacent to the UB, and the cortical interstitial stroma, which surrounds the CM (Fig. 1). Fate-mapping studies have demonstrated that the metanephric cells originate from the intermediate mesoderm, which also gives rise to the pronephros and mesonephros. Both largely regress in mammals but construct a functional kidney in lower vertebrates [1, 2].

After the establishment of the metanephros, the kidney organogenesis proceeds with iterative branching of the UB and subsequent differentiation of the nephrons from the CM adjacent to the tips of the UB. The differentiation of the nephrons from the progenitor cells begins as interplay between the CM and the UB leading to induction of the CM and subsequent transition of the induced cells into a pre-tubular aggregate, which undergoes a mesenchyme-to-epithelium transformation (MET) to establish a renal vesicle, which in turn becomes connected to the collecting duct system generated by the UB [3, 4].

**Fig. 2:**
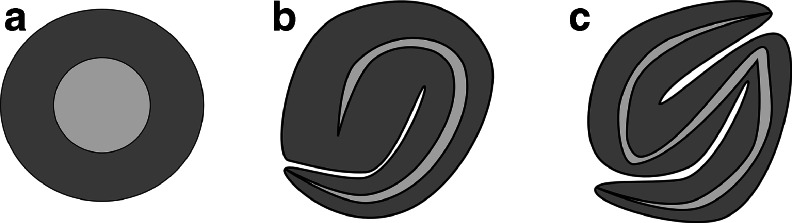
Nephron morphogenesis. As a consequence of mesenchyme-to-epithelium transition, a lumen containing renal vesicle (**a**) is assembled from the pre-tubular aggregate. A cleft forms within the epithelium of the renal vesicle turning it into a comma-shaped body (**b**). After another cleft formation step in the distal comma-shaped body an S-shaped body stage emerges (**c**). The distal part of the S-shaped body connects with the ureteric bud (UB)-derived collecting duct system while the proximal part forms the Bowman’s capsule

The renal vesicle matures into a functional nephron through comma- and S-shaped body stages (Fig. 2). The primordial nephron becomes elongated along the corticomedullary axis and forms morphologically distinguishable segments containing a Bowman’s capsule, a proximal convoluted tube, a loop of Henle, a distal convoluted tube, and a connecting tube. At the vascular pole, the nephron connects to the glomerular tuft composed of endothelial and mesangial cells, whereas the distal part joins with the collecting duct.

**Fig. 3:**
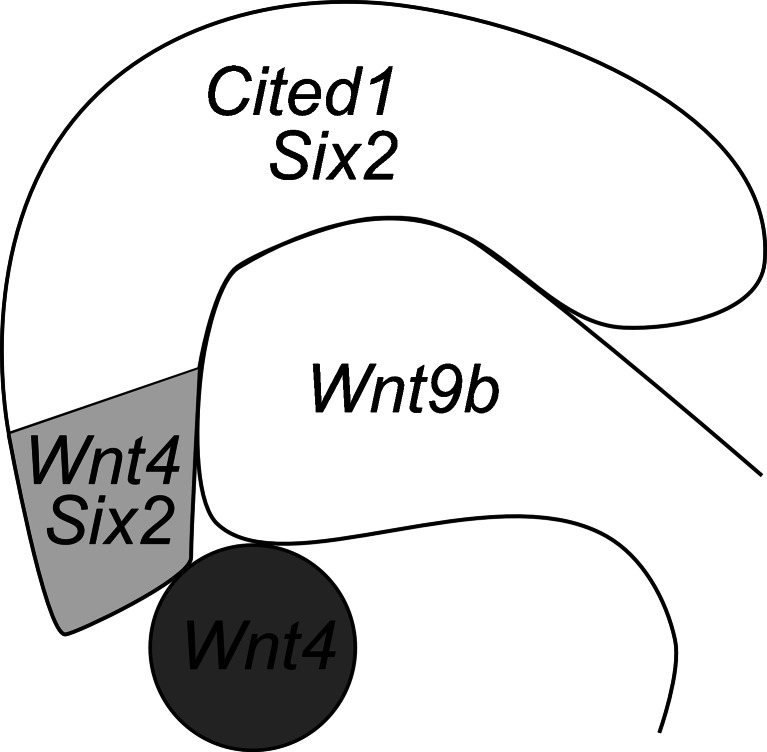
The relation between the ureteric bud (UB) tip and the cap mesenchyme (CM). The UB tip is surrounded by the adjacent cap mesenchyme (CM, *white and light grey*). *Wnt9b* is expressed in the UB. The CM consists of an uninduced and an induced CM highlighted by gene expression patterns. *Six2* is expressed in the whole CM, whereas only the uninduced CM (*white*) expresses *Cited1*. The induced CM (*light grey*) initiates *Wnt4* expression due to UB-derived Wnt9b. The induced CM transforms into a pre-tubular aggregate (*dark grey*) that separates from the CM. In the pre-tubular aggregate, *Six2* transcription is inhibited, whereas *Wnt4* expression is induced by auto regulation

Generation and analysis of several gene-targeted mouse models has identified elements of a transcriptional program behind nephrogenesis. The nephron stem cell pool marked by sine oculis-related homeobox 2 (Six2) maintains the putative self-renewal potential of these cells and prevents their premature epithelization [5, 6], but how is still poorly understood. Expression of *Six2* mRNA is rapidly down-regulated in the pre-tubular cell aggregates [7], while the respective protein persists in the proximal pole of the renal vesicle [8]. *Cited1* expression is down-regulated during the CM induction and thus *Cited1* specifically marks the dorsal un-induced CM that is still in the stem cell stage [7] (Fig. 3).

The Wnt signals play an important role in several distinct processes during kidney development. Evidence suggests that they are involved during initial induction of the CM and during the maturation of the nephron. Deficiencies in Wnt signaling may result in manifestations ranging from serious renal defects to more benign phenotypes.

The most thorough insight into vertebrate kidney development was obtained by using transgenic mice. These in vivo molecular genetic studies are in line with studies in the *Xenopus laevis* and of human diseases, collectively suggesting a conserved role for Wnt signaling in vertebrate kidney development. We review the literature covering the role of Wnt signaling in kidney development below.

## Induction of nephrogenesis by Wnt9b-mediated signaling

Contact between the UB and CM is required for successful kidney development. Reciprocal signaling between these tissues triggers the differentiation of the CM into a nephron. Separation of the UB from the MM results in regression of both tissues in culture [1, 9]. However, the isolated MM can be rescued from degeneration and induced to undergo nephrogenesis with exogenous signal sources.

When placed in contact with MM in culture, several embryonic tissues have the potential to provoke nephron differentiation in CM. The embryonic spinal cord (eSC) is the most potent one [10]. The capacity of the eSC to induce nephrogenesis is likely due to expression of Wnts [11], including Wnt4, which is a major endogenous Wnt present in induced CM [12]. Consistent with this, cell lines engineered to express other Wnts such as Wnt1, Wnt3a, Wnt4, Wnt7a, and Wnt7b were reported to trigger nephrogenesis in the isolated MM in a classical transfilter assay in which the MM and the Wnt-expressing cells are placed on the opposite sides of a porous polycarbonate filter [9].

Considerable effort was made to identify the UB-derived signal after the identification of Wnt4 as the major MM-derived nephrogenesis control signal [12]. Both inorganic and biological factors were found that were able to elicit nephrogenesis in a cultured MM, such as lithium ions [13] and leukemia inhibitory factor (LIF) secreted by the UB [14]. Although LIF was found to be UB derived, its deficiency did not lead to fatal phenotype [15], which would be expected if primary nephrogenic induction were impaired. Wnt9b was eventually identified as a primary UB-derived factor that is able to induce the CM to exit from the stem cell stage and become permanently committed to the nephron cell lineages [16].

The *Wnt9b* gene is expressed in the epithelial Wolffian duct prior to induction of metanephros development. *Wnt9b* expression continues in the UB where it is more pronounced in the stalk region than in the tips (Figs. 1 and 3). The expression of *Wnt9b* in the mice is maintained in the collecting ducts until adulthood [17]. Wnt9b-mediated induction in the CM initiates expression of *Wnt4*, *fibroblast growth factor 8* (*Fgf8*), *paired box 8 (Pax8)*, and *LIM homeobox protein 1 (Lhx1)*-encoding genes [12, 16, 18–20]. These genes fail to become expressed in the CM of *Wnt9b*-deficient embryonic kidneys and no nephrons form [16], as a result, *Wnt9b* knockout mice die soon after birth. It becomes apparent that Wnt9b-mediated signaling from the UB to the CM is essential for the primary induction of the CM.

## Wnt4 provides a key nephrogenesis control signal downstream from Wnt9b

The discovery that *Wnt4* function is necessary for formation of the pre-tubular aggregate and subsequent MET provided the first clue to the importance of Wnt signaling in kidney organogenesis [12]. Wnt9b-dependent *Wnt4* expression appears in the induced CM and persists until the S-shaped body stage [7, 12, 16] (Fig. 3). Fate-mapping studies indicate that mature nephrons form entirely from the progeny of the cells that have expressed the *Wnt4* gene [21]. Wnt4 function seems to be conserved because in the *Xenopus laevis xWnt4* is expressed in the pronephric anlage while later the expression becomes restricted to the tips of the pronephric tubule [22]. Similarly to the nephrons of mice, the pronephric tubules of* Xenopus* require *xWnt4* for their development [23].

Like *Wnt9b* deficiency, failure in Wnt4 signaling prevents nephrogenesis, and as a result only a vestigial kidney forms [12]. A mutation in the human *Wnt4* gene associates with renal degeneration indicating a clinical relevance of this signal [24, 25]. The *Wnt4* knockout is characterized by a failure in nephron differentiation, which halts prior to formation of the pre-tubular aggregates. This histological anomaly can be associated with a stage during which the endogenous *Wnt4* transcription is elevated. A non-functional *Wnt4* mRNA remains transcribed in other tissues in the *Wnt4* knockout model, but the expression is lost from the CM. This suggests an auto-inductive function of Wnt4 in the CM [12].

In the case of *Wnt4* deficiency, *Pax8* and *Fgf8* are initially expressed in few rudimentary pre-tubular aggregates [12, 20], which indicates that these factors function upstream of Wnt4 and downstream of Wnt9b (see above). Moreover, *Wnt4* is not detected in the metanephros of the *Fgf8*-null mice, whereas *Wnt4* expression remains in the *Lhx1*-deficient embryonic kidney [18–20] and *Lhx1* is absent in the kidney of both the *Fgf8-* and *Wnt4*-mutants. Collectively, the findings fit into a model in which, after the Wnt9b signaling, either Fgf8 and Wnt4 together or Wnt4 alone go on to induce the *Lhx1* gene in the pre-tubular aggregates. In any case, the Wnt4-dependent transcriptional program leading to *Lhx1* activation serves to differentiate the pre-tubular aggregate into the epithelialized renal vesicle. In short, Wnt4 acts in CM downstream of UB-derived Wnt9b and is necessary for the formation of the pre-tubular aggregate and subsequent epithelial derivatives.

## Wnt signal transduction during nephron induction

The downstream mechanisms elicited by Wnt signals in CM induction have been addressed in several studies. TCF-reporter mice lines *BAT-gal*, *TCF/Lef-lacZ, BATlacZ*, and *TCF-gfp* have demonstrated activation of the Wnt/TCF pathway in embryonic kidney [26–30]. These reporter lines revealed activity in the UB, but not in the MM and its derivatives [26, 29, 31–33]. However, another reporter line named *TCF/Lef-lacZ* [27] demonstrated TCF-mediated reporter activation in the distal S-shaped bodies and in the parietal epithelial layer of the Bowman’s capsule [34, 35], so some discrepancies exist between reporter line studies.

In contrast to results from the reporter lines, the Wnt/β-catenin/TCF pathway feedback target *Lef1* depicts the induced CM in a Wnt9b-dependent manner [7]. It is also noteworthy that the β-catenin-stabilizing small molecules (lithium chloride, LiCl, and 5′-bromoindirubin-3′-oxime, BIO), which both function by inhibiting GSK3β, induce nephrogenesis in the separated MM and also provoke BATlacZ activity [13, 33, 36, 37]. LiCl induces expression of the Wnt/β-catenin/TCF feedback genes *Lef1* and *Tcf1* and also *Wnt4* in the isolated MM [37]. It is reasonable to conclude that activation of the β-catenin/TCF signaling pathway is sufficient to act upstream or at the level of Wnt4 to elicit a nephrogenesis response.

Knockout of β-catenin function with *Six2Cre*-mediated recombination was reported to perturb the nephron differentiation [38]. Analysis of such deficient embryonic kidneys demonstrated markedly reduced expression of *Fgf8*, *Pax8*, *Wnt4*, and *Lhx1*, pointing to a failure in CM induction [38]. In contrast, β-catenin gain-of-function in the CM with a constitutively active β-catenin leads to ectopic CM induction and premature depletion of the nephron stem cell pool. These responses occur independently of Wnt9b and Wnt4 function [38]. Interestingly, the sustained activation of β-catenin blocks MET, suggesting that β-catenin-mediated signaling has to be inhibited to complete the nephron development. Activating β-catenin mutations have been reported in Wilms tumors, which typically show an incompletely epithelialized blastemal component indicating a failure during early nephron differentiation [39, 40].

Recent studies have revealed that stabilization of β-catenin with BIO up-regulates *Wnt4* and *Fgf8* and suppresses *Six2* expression in the aggregated MM cells [8]. Chromatin immunoprecipitation and sequencing indicated that the renal β-catenin protein binds to the* cis*-regulatory modules of the *Wnt4*, *Fgf8*, and *Six2* genes in the CM. These* cis*-regulatory elements also have TCF binding sites. In reporter studies of transgenic mice, the *Wnt4* and *Fgf8* *cis*-regulatory sequences driving β-galactosidase expression are able to promote the reporter expression when TCF sites are intact, whereas mutation of TCF motif impairs the β-galactosidase expression. This observation may be explained by β-catenin/TCF-mediated transcriptional up-regulation of the *Wnt4* and *Fgf8* genes in the CM derivatives. The observed reporter pattern by the *Six2* *cis*-regulatory module recapitulates the endogenous *Six2* expression even if the TCF motif is mutated. This indicates that the suppression of the Six2 associated with β-catenin accumulation is independent of the TCF motif [8]. In summary, β-catenin is sufficient to advance the nephron stem cell from their cell cycle to promote differentiation of the daughter cells towards the nephrons via up-regulation of *Fgf8* and *Wnt4*. This induction stage is apparently controlled by Wnt9b via the β-catenin/TCF/Lef pathway and TCF/Lef-independent β-catenin-mediated transcriptional program regulation.

## Downstream transduction of Wnt4 signaling

After induction of the CM by Wnt9b, Wnt4 functions to promote MET [12]. Because the epithelization is inhibited by aberrant β-catenin signaling [38] and various TCF reporter lines report negative activity in CM, alternative modes of Wnt4 signal transduction have been under scrutiny. Indeed, the Wnt/calcium/NFAT (nuclear factor of activated T cells) pathway may function as a downstream effector for Wnt4 signaling in the pre-tubular cell aggregates [32, 33]. Cytoplasmic calcineurin-dependent NFAT factors are expressed in early nephron forming cells, while the inhibitor for calcineurin inhibits nephrogenesis. Moreover, experimentally increased intracellular calcium in part rescues the *Wnt4*-deficency phenotype by correcting failure in nephrogenesis [32]. Wnt4 can also induce a calcium influx and calcium/calmodulin-dependent protein kinase II phosphorylation in primary MM cells [33]. This evidence suggests that Wnt4 induced by Wnt9b/β-catenin functions in the CM at least in part via the calcium/NFAT pathway to promote MET. Lithium and BIO function probably upstream of Wnt4 via β-catenin stabilization, whereas the calcium ionophores, which induce intracellular calcium concentration, may mimic the action of Wnt4 to promote nephrogenesis.

## Role of the planar cell polarity pathway in control of nephron maturation

After the MET, the presumptive nephron becomes segmented and aligned into a unique corticomedullary oriented structure. Such complex architecture with defined tube length and diameter requires oriented cell division and coordinated cell motility. The Wnt signals have been shown to control these processes and also when deregulated to lead to pathophysiological changes and cystic kidney disease [41].

After the induction of the CM, Wnt9b also has a role during later nephrogenesis in the coordination of the planar cell polarity and in cell intercalation during tubulogenesis via a process of convergent extension [17, 42]. Still, after birth, Wnt9b signaling has a role for oriented epithelial cell divisions to regulate the diameter of the epithelial tubules [17]. Renal cysts in *Wnt9b*-hypomorph mice are derived from the proximal convoluted tubules and attributed to disturbed activation of the Rho family GTPase (Rho) and Jun kinase, which are components of the Wnt/planar cell polarity (PCP) pathway [17].

Knockdown of Wnt/PCP components in frogs, such as *dishevelled associated activator of morphogenesis 1* (*Daam1*), *Rho guanine nucleotide exchange factor* (*WGEF*), and *Rho*, consistently impairs the late morphogenesis of the pronephric tubules and duct. The pronephic cells of such morphants retain their ability for differentiation. This indicates that the Wnt/PCP pathway plays a role after the MET [43]. Studies of the tubular elongation in the *Xenopus* nephron with the time-lapse approach indicated convergent extension-type cellular movement during nephron maturation [42]. The process involves an evolutionarily conserved rosette-based cell organization. This is dependent on Wnt9b in mice and dishevelled-2 in the *Xenopus* [42]. It is reasonable to conclude that the Wnt/PCP signaling regulates nephron maturation after the initial induction and the stages of MET.

## The Wnt/β-catenin pathway in nephron maturation

Given that the Wnt/PCP pathway becomes important in nephron maturation and that the Wnt/β-catenin signal transduction pathway needs to be suppressed to accomplish MET, mechanisms balancing the downstream signaling had to be explored. In addition to failure in MET and Wilms tumor association, incorrect activation of β-catenin signaling has been shown to cause renal cyst formation [44, 45], raising the possibility that cystic renal disease models may elucidate the factors behind downstream control of Wnt signaling in nephron maturation. A mouse line modeling nephronophthisis [46], a ciliopathy presenting with cystic renal disease, has provided a primary cilium protein named inversin as a potential Wnt signal transduction regulator candidate [47]. Inversin was shown to reduce the β-catenin amount by increasing proteasomal degradation of cytoplasmic dishevelled. Furthermore, inversin was demonstrated to promote β-catenin-independent Wnt signal transduction [47]. Finally, it has been speculated that the urine flow in early tubules may regulate the cellular response to Wnt signals via the primary cilium. Indeed, exposure of flow primary cells derived from the inner medullary collecting ducts increases inversin but decreases the level of β-catenin [47]. Another mechanism coordinating Wnt/b-catenin signaling inhibition functions after the renal vesicle stages and involves protein kinase A [48].

In addition to mutual organization, well-controlled differentiation of the nephron epithelium into highly specialized cell types is critical for kidney function. The specification of the proximodistal segmentation begins relatively early in morphogenesis as depicted by polarized expression of certain genes in the assembled renal vesicle [4]. The presumptive nephron contains progenitors for the parietal and visceral layers of the Bowman’s capsule already during the S-shaped body stage [34]. β-catenin appears to be coupled to this control step as well, since conditional loss of its function in the nephron progenitors after the pre-tubular aggregate stage switches the fate of the parietal epithelial cells into the visceral phenotype presenting capillary connections [34]. Collectively, it appears that the Wnt signaling pathway is important during later nephron maturation stages after the MET. However, the downstream signal transduction involves suppression of the β-catenin branch and acquired dominance of the Wnt/PCP pathway. The switch between these pathways may be carried out by changes in the responsiveness of the tubular cells to endogenous Wnt ligands.

## Wnt11/GDNF signaling loop in control of the collecting duct system development

The iterative UB bifurcation is a key determinant of the final nephron number and therefore of the size of the adult kidney. Glial cell line-derived neurotropic factor and Ret proto-oncogene (GDNF/Ret)-dependent signaling promotes formation of the UB from the Wolffian duct and subsequent branching during generation of the kidney collecting duct system. From the initial stages of kidney development, the CM expresses GDNF that signals to the UB via Ret to promote UB ingrowth and branch formations (Fig. 1). Deficiencies in either of these factors compromise kidney development due to growth failure of the UB [49–51].

Wnt11 functions with the GDNF/Ret to promote collecting duct development [52]. The *Wnt11* gene is expressed at the UB tips [53, 54] (Fig. 1). Unlike Wnt1, Wnt3a, Wnt4, Wnt7a, and Wnt7b, Wnt11 fails to induce nephrogenesis in the MM explants [9]. Amphibian *Wnt11b*, which mammals have lost, can induce nephrogenic in the *Xenopus* pronephros [55]. As a result, it has been suggested that the loss of *Wnt11b* during evolution may be behind the regression of the mammalian pronephros [55]. *Wnt11* deficiency in the mouse reduces kidney size and diminishes the number of nephrons [52]. The lack of *Wnt11* function delays the first trifurcation event of the UB and was associated with a transient reduction in GDNF expression in the CM. In turn, exogenous GDNF promotes *Wnt11* expression in the UB tips of isolated metanephric explants, supporting the finding that GDNF and Wnt11 are interdependent [56]. This notion is further supported by the findings that the few *Ret* homozygous mutant embryos that form the T-stage UB fail to express *Wnt11* in the UB tips [52]. Furthermore, on a *Ret* heterozygous background the *Wnt11* knockout allele reduces the number of nephrons dose-dependently [52]. In mice, it appears Wnt11 and the GDNF/Ret pathways form a reciprocally positively regulated signal loop. This contributes to the generation of a sufficient number of UB branches to form the collecting duct system and to induce a corresponding amount of nephrons.

## The frizzled receptors in the kidney organogenesis

Even though the essential role of the Wnt ligands in kidney organogenesis is well demonstrated, we have very limited knowledge of their receptors. Of the frizzled receptors at least *Fz2, Fz4, Fz6, Fz7, Fz8*, and *Fz10* are expressed in mouse embryonic kidney [57, 58]. In *Xenopus*, morpholino-mediated knock down of *Xfz8* impaired the epithelial morphogenesis of the pronephric duct and tubules [59].

In the mouse, compound *Fz4* and *Fz8* knock out allows the nephron to segment but the kidney size is reduced due to diminished proliferation and UB branching which resembles the phenotype of the *Wnt11* knockout mice [52, 58]. The *Fz8* knockout reduces the kidney size only when *Fz4* function is impaired, indicating some redundancy between the frizzleds. Wnt11 may signal via fz4 since this combination activates the TFC/Lef and the Rho reporters in cell culture. Concurrent expression of Wnt11 and Fz8 activates Rho suggesting specific mode of signaling via the frizzled subclasses [58]. Thus, Fz4 and Fz8 may act in synergy to transduce Wnt11 signaling and promote UB branching.

## Kidney medulla development involves Wnt7b and Wnt4 function

In regard to kidney function, plasma filtration occurs in the cortex, while the osmoregulation of the filtrate occurs in the medulla where the Henle’s loops and collecting ducts are located. The radially defined regions provide the foundations for the kidney to perform its secretory and excretory functions.

Of the *Wnt* genes*, Wnt7b* expressed in the stalk of the UB [16, 53], and Wnt7b-expressing cells can induce nephrogenesis in the separated MM tubule induction assay [9] (Fig. 1). However, knock out of *Wnt7b* in the UB cells leaves the nephrogenesis zone unaffected [60], whereas the medulla degenerates. In the *Wnt4-*, *Fgf8-*, and *Lim1*-deficiency perturbing nephrogenesis, a well-distinguishable medullary still develops [12, 18–20]. It appears medulla formation is independent of the nephron formation process. This conclusion is further supported by the finding that the size of the *Wnt7b*-deficient kidney remains close to normal and the number of UB tips remains unaffected. Consistent with the localization of *Wnt7b* mRNA, segmentation of the cortical nephrons occurs normally and the proximal and distal convoluted tubules and podocytes develop. However, the loops of Henle in the medulla are truncated without Wnt7b signaling. The proximal UB is also dilated, which can be attributed to changes in oriented cell divisions in the UB. Consistently, a deficiency in Wnt antagonist *dickkopf-1* in the UB and developing nephrons leads to overgrowth of the renal medulla, suggesting that medullary development depends on a balance between Wnt7b and its antagonist dickkopf-1 [61].

In addition to the CM, the *Wnt4* gene is expressed in the medullary stroma during kidney development. Here Wnt4 expression is not auto-regulated [53, 62]. *Wnt4* knock out, however, reduces stromal *Bmp4* expression and leads to absence of α-smooth muscle actin-expressing cells, suggesting a failure in pericyte differentiation. This evidence suggests Wn7b and Wnt4 are utilized as coordinating signals in developing kidney medulla.

## Downstream Wnt signaling factors in medulla development

Nuclear translocation of β-catenin in embryonic kidney has been initially reported in human tissue [63]. Nuclear β-catenin is intense in the medullary stromal cells surrounding the collecting ducts of fetal human kidney. It turned out that the medullary-located periductal cells also express the *Axin2* and Lef1 Wnt signaling feedback targets [60]. Their expression is Wnt7b dependent, suggesting a response to the Wnt signaling [60]. Moreover, BAT-gal reporter demonstrated colocalization with Lef1 expression [60]. These observations can be considered highly suggestive for Wnt/β-catenin/TCF pathway operation in embryonic kidney medullary cells. Consistent with this possibility, the inactivation of β-catenin function from the stromal cells with the aid of *FoxD1Cre*-mediated recombination generates a similar phenotype as that of *Wnt7b* knockout [60]. From this, we can conclude that the reciprocal interaction between the collecting ducts and the kidney stroma involves Wnt/β-catenin/TCF signaling, and this in turn coordinates the renal medulla development.

## Outlook for the future

Wnt signaling is critical for several aspects of kidney organogenesis and it highlights the context-dependency of the downstream signaling systems. The key role of Wnt signaling in nephron induction and differentiation is well established, but the later morphogenic events that involve the PCP pathway need to be revealed in more detail. Given the severe phenotypes generated by the Wnt signaling system knock outs, failures in Wnt signal transduction will likely be associated with human congenital anomalies of the kidney and urinary tract (CAKUT). We expect to find more direct links between the renal Wnt signaling pathway and genetic diseases, which may lead to the identification of new signal transduction components and mechanisms. Of particular interest are the primary cilium and its role in regulating the responses to Wnt ligands in the tubular cells. Due to often-subtle phenotype differences of the mutations that affect later stages of kidney organogenesis, novel methods such as three-dimensional imaging and time-lapse techniques will aid the analysis.

There are Wnt ligands, such as Wnt2b, Wnt5a, and Wnt6, that are expressed in the embryonic kidney but of which the role remains open [64, 65]. Wnt2b does not appear to be essential for tubule induction, since Wnt2/Wnt2b double knockout mice do not develop overt kidney phenotypes [66]. However, Wnt2b may function as a stromal signal to fine-tune UB morphogenesis [64, 67]. Wnt6 expression is detected in the UB and it has the capacity to induce nephron differentiation in the isolated MM [65], but no kidney-related phenotypes have been reported in the Wnt6-deficient mice.
